# Activation mechanisms of the Hippo kinase signaling cascade

**DOI:** 10.1042/BSR20171469

**Published:** 2018-08-29

**Authors:** Sung Jun Bae, Xuelian Luo

**Affiliations:** 1 Department of Pharmacology, University of Texas Southwestern Medical Center, Dallas, TX 75390, U.S.A.; 2 Department of Biophysics, University of Texas Southwestern Medical Center, Dallas, TX 75390, U.S.A.

**Keywords:** Hippo pathway, phosphorylation, phosphatase, Signal transduction, tumor suppressors

## Abstract

First discovered two decades ago through genetic screens in *Drosophila*, the Hippo pathway has been shown to be conserved in metazoans and controls organ size and tissue homeostasis through regulating the balance between cell proliferation and apoptosis. Dysregulation of the Hippo pathway leads to aberrant tissue growth and tumorigenesis. Extensive studies in *Drosophila* and mammals have identified the core components of Hippo signaling, which form a central kinase cascade to ultimately control gene expression. Here, we review recent structural, biochemical, and cellular studies that have revealed intricate phosphorylation-dependent mechanisms in regulating the formation and activation of the core kinase complex in the Hippo pathway. These studies have established the dimerization-mediated activation of the Hippo kinase (mammalian Ste20-like 1 and 2 (MST1/2) in mammals), the dynamic scaffolding and allosteric roles of adaptor proteins in downstream kinase activation, and the importance of multisite linker autophosphorylation by Hippo and MST1/2 in fine-tuning the signaling strength and robustness of the Hippo pathway. We highlight the gaps in our knowledge in this field that will require further mechanistic studies.

## Introduction

The Hippo pathway controls cell numbers and organ size in multicellular organisms through restricting cell growth and proliferation and promoting apoptosis. It plays important roles in organ development, tissue regeneration, and stem cell maintenance. Dysregulation of the Hippo pathway is implicated in a wide range of human cancers [1–4]. Targetting this pathway with small molecules may lead to novel therapeutic interventions for the treatment of human diseases [4,5].

**Figure 1:**
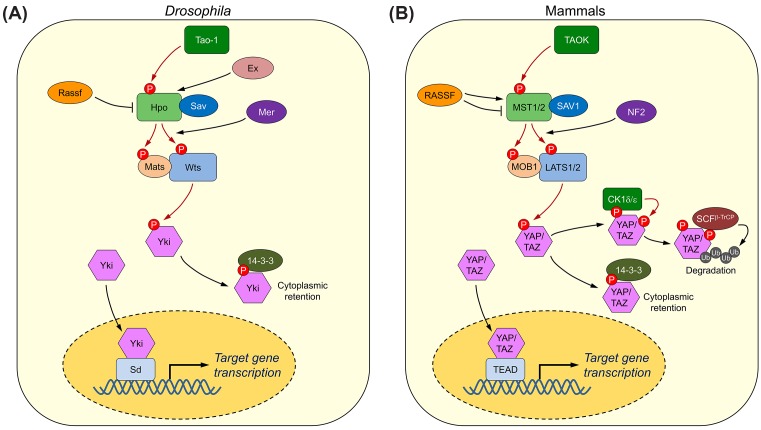
The conserved Hippo signaling network. The core Hippo pathway in *Drosophila* (**A**) and mammals (**B**). The core Hippo signaling events are shown. The same color scheme is used for the corresponding pathway components. Phosphorylation events are indicated by dark red arrows. See the text for further details.

The core components of this pathway were initially identified by genetic screens seeking overgrowth phenotypes in *Drosophila*. The name ‘Hippo’ came from the fly phenotype of one core component [6–9]. Subsequent biochemical, cellular, and genetic studies in both *Drosophila* and mammals have established that these core components are highly conserved, and constitute the central signaling node of the Hippo pathway [2,10–17] (Figure 1). In this review, we focus on the core machinery of the mammalian Hippo pathway, and refer to the nomenclature of *Drosophila* homologs in specific context as needed.

The core of the mammalian Hippo pathway contains a kinase signaling cascade, formed by two types of serine/threonine kinases – Mammalian Ste20-like 1 and 2 (MST1/2; the Hippo homologs) and Large Tumor Suppressor 1 and 2 (LATS1/2), and two adaptor proteins – the WW-domain containing scaffold protein Salvador (SAV1) and the Mps One Binder 1 (MOB1). Multiple upstream signals activate the MST–LATS kinase cascade to regulate the localization of two oncogenic transcriptional co-activators, the Yes-associated protein (YAP) and the transcriptional co-activator with PDZ-binding motif (TAZ) [18–22]. When the Hippo pathway is turned on, the upstream MST1/2 kinases in complex with SAV1 phosphorylate and activate the LATS1/2–MOB1 complexes. Activated LATS1/2–MOB1 then phosphorylate YAP/TAZ, resulting in their cytoplasmic retention by 14-3-3 proteins [23–27]. LATS1/2-mediated phosphorylation also triggers subsequent phosphorylation of YAP/TAZ by casein kinase 1δ/ε, leading to their SCF^β-TrCP^ E3 ligase-induced degradation (Figure 1B) [28,29]. Reduced nuclear YAP/TAZ levels consequently lead to the down-regulation of the downstream targets of the Hippo pathway. When the Hippo signaling is turned off, unphosphorylated YAP/TAZ translocate into the nucleus and form functional transcriptional complexes with TEA domain proteins 1–4 (TEAD1–4) [27,30]. YAP/TAZ–TEADs promote the expression of Hippo-responsive genes that are involved in cell proliferation, migration, and survival (reviewed in [2,11,31]).

Extensive studies have revealed a myriad of intrinsic and extrinsic signals that can activate the Hippo pathway, including cell–cell contact, stiffness of the extracellular matrix, stress signals, and cell polarity (reviewed in [2,11,32–34]). These signals mainly modulate phosphorylation events of the core MST–LATS kinase cascade through upstream or peripheral components of the pathway. For example, in *Drosophila*, two FERM domain-containing proteins, Merlin (Mer) and Expanded (Ex), form spatially distinct complexes with other Hippo regulators at the cell cortex or intercellular junctions to integrate upstream signals for the activation of the core Hippo cascade [18,35,36]. In mammals, the Neurofibromatosis type 2 (NF2) protein, the mammalian homolog of Mer, promotes Hippo signaling through directly binding and recruiting the effector kinase LATS to the plasma membrane and thereby enhancing its phosphorylation by the membrane-associated MST–SAV1 kinase complex [33,37,38]. The phosphorylation-mediated regulation of the Hippo core kinase cascade is further complicated by cross-talk between the Hippo pathway and several other developmental signaling pathways, such as Hedgehog, Wnt, and Notch pathways [31].

Although numerous studies have identified the core components of the Hippo pathway and revealed their cellular functions, understanding of their interactions at the molecular level has lagged behind. Recent mechanistic and structural studies have begun to fill this gap of our knowledge by elucidating the molecular mechanisms of the phosphorylation-dependent regulatory events during the activation of the core MST–LATS kinase cascade. In this review, we summarize the activation mechanisms of the Hippo core kinase cascade and highlight open questions in this area that require further mechanistic studies.

## Regulation of MST1/2 autoactivation by SAV1/RASSF/Hippo-mediated dimerization

**Figure 2:**
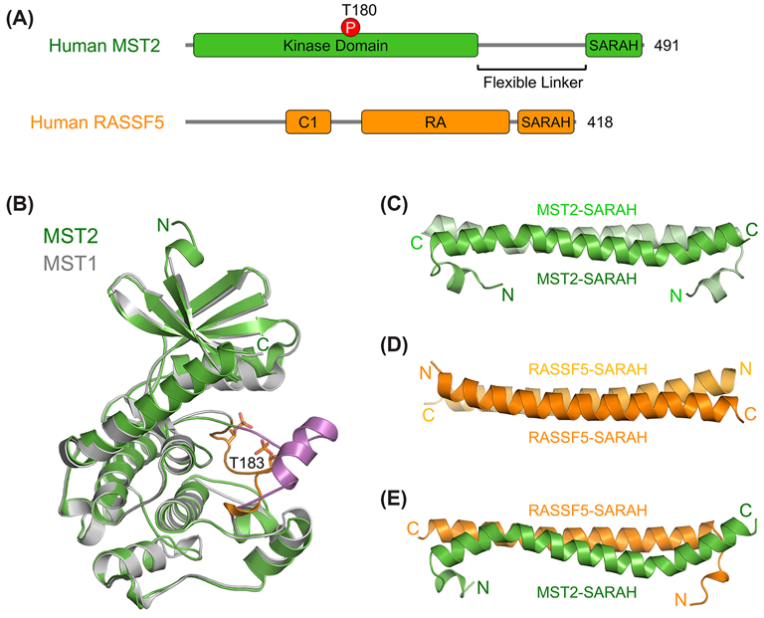
Structural basis for SARAH-mediated MST1/2 kinase autoactivation (**A**) Domain organization of human MST2 and RASSF5. (**B**) Structure superposition of the kinase-active MST1 kinase domain (PDB ID: 3COM; deposited by New York SGX Research Center for Structural Genomics) and the kinase-inactive MST2 kinase domain (PDB ID: 4LG4). MST1 is colored gray, and MST2 is colored green. The activation loop from MST1 is colored orange with residue p-T183 shown in sticks. The activation loop from MST2 is colored magenta. (**C**) The crystal structure of human MST2 SARAH homodimer (PDB ID: 4OH9). Protomer A is colored green and protomer B is colored light green. (**D**) The crystal structure of murine RASSF5 homodimer (PDB ID: 2YMY). Protomer A is colored orange and protomer B is colored light orange. (**E**) The crystal structure of human MST2–RASSF5 SARAH heterodimer (PDB ID: 4LGD). RASSF5 is colored orange and MST2 is colored green. All structural figures were generated with PyMol (https://www.pymol.org). Abbreviation: RA, Ras-associated domain.

Activation of the MST1/2 kinases is a key initiating event in Hippo signaling. Understanding the molecular basis of this activation is of critical importance in the field. MST1/2 contain an N-terminal kinase domain, a C-terminal SARAH (Salvador/Ras-association domain family (RASSF)/Hippo) domain, and a flexible linker that connects these two domains (Figure 2A). The crystal structures of both active MST1 kinase domain (with its activation loop phosphorylated) and MST2 kinase domain in its inactive, unphosphorylated state have been determined (Figure 2B) [39]. The kinase domain in both structures adopt a canonical kinase fold with a bilobed architecture. The overall structures of MST1 and MST2 kinase domains are very similar with an RMSD of 1.0 Å excluding the activation loop (T-loop). However, structural comparison of the inactive and active states of MST1/2 kinase domains reveals prototypical phosphorylation-dependent conformational changes in the T-loop. Phosphorylation of T183 in the MST1 activation loop allows it to adopt an extended conformation suitable for substrate binding. By contrast, the unphosphorylated activation loop in the inactive MST2 forms an α-helix that blocks the kinase active site. Therefore, MST1/2 activation requires a phosphorylation-mediated conformational rearrangement at the activation loop (T183 for MST1 and T180 for MST2). Recombinant MST1/2 purified from bacteria forms dimers and are phosphorylated at the activation loop and fully active, indicating that MST1/2 can be autoactivated by trans-autophosphorylation through homodimerization without the requirement of upstream kinases [39]. On the other hand, *Drosophila* Tao kinase 1 (Tao-1) and its mammalian orthologs TAOK1-3 have been reported to directly phosphorylate Hippo/MST1/2 at the T-loop to activate the kinase, suggesting that Tao-1/TAOK might be an upstream kinase for Hippo/MST1/2 under certain conditions (Figure 1) [40,41]. It is not clear how Tao-1/TAOK-mediated Hippo/MST1/2 activation is regulated, although some studies suggest that Tao-1/TAOK might modulate the effect of Mer/NF2 and Ex on the Hippo pathway.

The C-terminal SARAH domain of MST1/2 is a coiled-coil domain that mediates the constitutive homodimerization of MST1/2 [42]. Autophosphorylation of full-length MST2 is much more efficient than that of kinase domain alone, indicating that SARAH-mediated homodimerization of MST2 is critical to orient the two kinase domains in optimal position for their trans-autophosphorylation and activation [39,42,43]. Because MST1/2 have this intrinsic ability of autoactivation through dimerization, their kinase activity needs to be tightly regulated by other regulators to prevent improper Hippo signaling and developmental defects. One mechanism to prevent Hippo/MST1/2 autoactivation is to disrupt their SARAH-mediated dimerization [44]. The RASSF tumor suppressor proteins, effectors of Ras, are important MST1/2 regulators [45,46]. RASSFs also contain a C-terminal SARAH domain which can form a heterodimer with the MST1/2 SARAH domain (Figure 2A) [47].

Structures of the MST1/2 SARAH homodimer, the RASSF5 SARAH homodimer and the MST1/2–RASSF5 SARAH heterodimer have been determined (Figure 2C–E) [39,47–49]. Both SARAH homodimer and heterodimer form a long antiparallel coiled coil. The RASSF5 SARAH homodimer is much less stable than the MST1/2 SARAH homodimer. The SARAH heterodimer has higher affinity. Thus, RASSF5 SARAH can disrupt the MST1/2 SARAH homodimer. The dimer interfaces of MST1/2 SARAH to RASSF5 SARAH or to another MST1/2 SARAH are essentially identical, readily explaining how RASSF5 binding can disrupt SARAH-mediated MST1/2 homodimerization. More importantly, recombinant RASSF5 SARAH can suppress MST1/2 trans-autophosphorylation, indicating that disrupting MST1/2 SARAH homodimer is sufficient to block kinase autoactivation. Interestingly, binding of RASSF5 to the already activated MST1/2 does not inhibit their kinase activities, and may stimulate MST1/2 function through other mechanisms [39].

The roles of RASSFs in the tumor-suppressive Hippo pathway are far from clear, however. As a negative regulator of the Hippo pathway, both *Drosophila* and mammalian RASSFs can form SARAH–SARAH heterodimers with Hippo/MST1/2, thereby inhibiting Hippo kinase autoactivation [39,50–53]. On the other hand, RASSFs can positively regulate MST1/2 [54–59]. For example, co-expression of RASSFs with Ras enhances the MST1 kinase activity *in vivo* [52]. RASSF1A promotes MST1/2 phosphorylation and activation through preventing MST1/2 dephosphorylation by the phosphatase PP2A [60]. Therefore, through spatial and temporal regulation, RASSFs appear to have dual functions in Hippo signaling in response to diverse cellular signals.

## Mechanism of MOB1-dependent LATS1/2 activation by MST1/2

**Figure 3:**
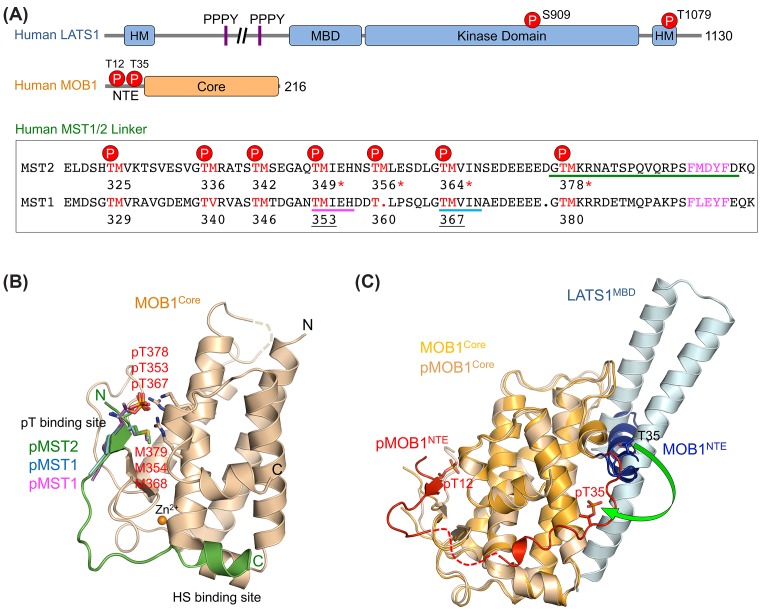
Structure basis for MOB1-dependent LATS1 activation by MST2 (**A**) Domain organization of human LATS1 and MOB1. The MST1 and MST2 linker sequences are shown with seven autophosphorylation TM colored in red. The hydrophobic sequence (HS) is colored magenta. The four pTM sites of MST2 that bind to MOB1 are indicated by red asterisk. The two pTM sites of MST1 that are shown in (**B**) are underlined. (B) Structure superposition of human MOB1 in complex with the pMST2 linker peptide (PDB ID: 5BRM), human MOB1 bound to MST1 pT353 phosphopeptide (PDB ID: 5TWG), and human MOB1 bound to MST1 pT367 phosphopeptide (PDB ID: 5TWH). Only pMST2 bound MOB1 is shown for clarity. MOB1 is colored wheat, and pMST2 is colored green. MST1 pT353 is colored magenta, and MST1 pT367 is colored cyan. The amino acid sequences of three MST1/2 phosphopeptides in the structures are indicated with corresponding colored lines in (A). Residues pT378, pT353, pT367, M379, M354, and M368 are shown as sticks. (**C**) Structure superposition of unphosphorylated MOB1 (PDB ID: 5B5V) and pMOB1–LATS1 (PDB ID: 5BRK). Unphosphorylated MOB1 core is colored gold, and MOB1 NTE is colored dark blue. The pMOB1 core is colored wheat, and pMOB1 NTE is colored red. The missing residues are connected by dashed line. LATS1 MBD is colored light cyan. The phosphorylation-dependent movement of MOB1 residue T35 is indicated by a green arrow. Abbreviations: NTE, N-terminal extension; pMST2, pT378 MST2 linker peptide.

LATS1/2 belong to the NDR family of kinases, a subgroup of the AGC kinase family [61]. LATS1/2 contain a MOB-binding domain (MBD), a C-terminal kinase domain, and a hydrophobic motif (HM) at the C-terminus (Figure 3A). Similar to other NDR kinases, LATS1/2 kinase activation requires two sequential phosphorylation steps [62–65]. First, LATS1/2 are phosphorylated by upstream kinases MST1/2 at a highly conserved threonine residue in HM (T1079 for LATS1). Second, phosphorylated HM allosterically triggers autophosphorylation of the LATS1/2 T-loop (S909 for LATS1) and thus fully activates these kinases. Both phosphorylation events of the HM and T-loop are essential for LATS1/2 kinase activation [62,66]. Recent studies show that the MAP4K family kinases can also phosphorylate LATS1/2 at their HM and fully activate the kinase in parallel to MST1/2 [67–70]. We will focus our discussion on the canonical Hippo pathway, since the activation of the MST–LATS kinase cascade is sufficient to inhibit YAP/TAZ-mediated gene expression [71,72].

The evolutionarily conserved MOB proteins function as important kinase regulators from yeast to man. Amongst human MOB proteins (hMOB1A/B, hMOB2, hMOB3A/B/C, and hMOB4), only MOB1 functions as an activator of LATS1/2 kinases [73,74]. MOB1 is thus the mammalian homolog of *Drosophila* Mats. It contains a flexible N-terminal extension (NTE) and a C-terminal globular core domain (Figure 3A). MST1/2 phosphorylate MOB1 at residues T12 and T35 in NTE [75]. p-MOB1 then binds to the conserved MBD of LATS1/2 and promotes kinase activation [64,76]. Phosphorylation of MOB1 NTE by MST1/2 is critical for the formation of the LATS1/2–MOB1 complex *in vitro*, as the T12A/T35A (2TA) mutant of MOB1 no longer binds to LATS1/2 [75].

Recent biochemical and structural studies have revealed the molecular basis of MOB1 phosphorylation by MST1/2 [77]. As shown by NMR spectroscopy, active MST2 autophosphorylates multiple residues in its linker, creating phospho-docking motifs for MOB1. Phosphorylation of MOB1 by MST2 requires its binding to the autophosphorylated linker of MST2. The crystal structure of MOB1 bound to a pT378 MST2 linker peptide (pMST2) revealed that MOB1 associates with pMST2 through two spatially separated binding sites, the p-threonine (pT) binding site and the hydrophobic sequence (HS)-binding site (Figure 3B) [77]. At the pT site, pT378 binds to a highly positively charged pocket while M379 inserts into an adjacent hydrophobic pocket on MOB1. Therefore, the major interactions at this site involve the pTM dipeptide motif of pMST2, similar to those observed in the crystal structure of human MOB1 bound to an optimized yeast Nud1-like phosphopeptide [78]. At the HS site, the FMDYF HS of pMST2 folds into a short helix and binds to a shallow groove on MOB1. The loop between the pTM and HS motifs is disordered. Taken together, pMST2 engages MOB1 at two distinct sites through pTM and HS bipartite interactions.

There are seven autophosphorylated TM motifs in the human MST2 linker (Figure 3A) [77]. Intriguingly, four pTM motifs (T349, T356, T364, and T378) are functional in MOB1 binding, indicating that these sites act redundantly to bind the pT-binding site of MOB1. A common HS motif complements each pTM motif to enhance MOB1 binding to the phosphorylated MST2 linker. This redundancy might provide more robust MOB1 activation or fine-tune downstream signals in response to certain upstream Hippo signals. A recent proteomics and biochemical study shows that multiple phospho-sites in the human MST1 linker region provides redundancy in MOB1 binding (Figure 3A), and the substrate consensus of MST1/2 kinase closely matches that of the phosphopeptide-binding consensus of MOB1 [79]. Thus, autophosphorylation of the MST1/2 linker creates specific MOB1 docking sites. This is further supported by the crystal structures of human MOB1 bound to human MST1 pT353 and pT367 phosphopeptides, which demonstrate that MOB1 recognizes each individual pT site in the MST1/2 linker through sequence-specific binding to its pT-binding site (Figure 3B). Importantly, this study also reveals that the phosphopeptide-binding function is shared amongst all but one of the human MOB proteins, strongly suggesting that MOB-mediated phospho-recognition plays an important role in their diverse cellular functions.

Only p-MOB1 can efficiently bind to LATS1 *in vitro*, indicating that MOB1 NTE undergoes a phosphorylation-dependent conformational change, which allows MOB1 to switch from a closed state to an open state for LATS1 binding. Indeed, several recent structures have provided structural snapshots of the phosphorylation-dependent switch-like behavior of MOB1 NTE [77,80,81]. Due to its flexible nature, previous structural studies of MOB1 have excluded the NTE region to facilitate crystallization [82]. The crystal structure of p-MOB1 in complex with a LATS1 fragment containing MBD has provided the first full-length MOB1 structure in its open conformation (Figure 3C) [77]. In the structure, p-MOB1 NTE associates intramolecularly with the MOB1 core. Phospho-T12 (pT12) binds to the pT-binding site of MOB1. The neighboring residues of pT35 occupy the HS-binding site. The LATS1 MBD folds into two antiparallel helices and binds to an acidic patch on MOB1. The LATS1-binding and pMST2-binding surfaces of MOB1 do not overlap, consistent with the observation that LATS1 and MST2 can bind to MOB1 simultaneously *in vitro*. Moreover, a recent crystal structure of a budding yeast NDR kinase and MOB complex reveals that this type of binding mode between LATS1 and MOB1 is conserved in the NDR family of kinases [83].

**Figure 4:**
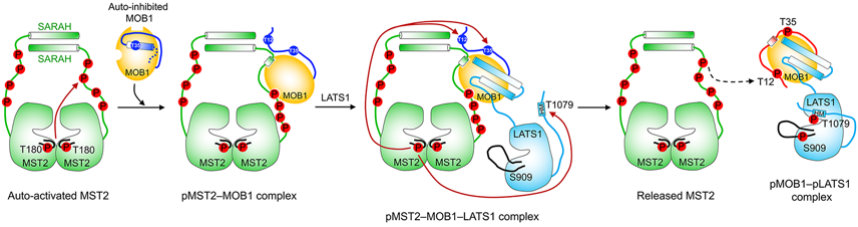
Model for the interdependent, multistep phosphorylation events by upstream kinase MST2 to activate the downstream kinase LATS1 through MOB1 dynamic scaffolding and allosteric activation mechanisms. Key phosphorylation events are indicated by dark red arrows. See the text for further details.

Intriguingly, deletion of the N-terminal 15 residues (including T12) of MOB1 enables its binding to LATS1, suggesting that the NTE of MOB1 blocks LATS1 binding by adopting an autoinhibited conformation, which can be disrupted by MST2-mediated phosphorylation of T12 [77]. Indeed, a recently determined crystal structure of full-length unphosphorylated MOB1 shows that residues 20–39 of MOB1 NTE (including T35) folds into a ‘switch-like’ helix that blocks the LATS1-binding surface of MOB1 intramolecularly (Figure 3C) [81]. Although the very N-terminal region (residues 1–15) of MOB1 does not directly associate with the LATS1-binding surface in the structure, it might stabilize the interaction between the ‘switch-like’ helix and the LATS1-binding surface. Phosphorylation of MOB1 T12 and T35 by MST1/2 destabilizes these interactions and promotes the dissociation of the ‘switch-like’ helix from the LATS1-binding surface, thus enabling LATS1 binding to MOB1. Therefore, the NTE of MOB1 acts as a phosphorylation-mediated molecular switch to dictate the binding between MOB1 and LATS1/2 in the Hippo pathway.

p-NTE of MOB1 occupies the same binding surface of MOB1 as that of pMST2. p-NTE of MOB1 thus competes with pMST2 for MOB1 binding. This competition is expected to hinder the formation of the pMST2–MOB1–LATS1 ternary complex and prevents efficient LATS1 phosphorylation by MST2. Thus, the phosphorylation of MOB1 NTE by MST2 must be temporally regulated. Indeed, binding of pMST2 to unphosphorylated MOB1 is sufficient to shift the conformational equilibrium of MOB1 from the closed state to the open state, enabling LATS1 binding and the formation of the pMST2–MOB1–LATS1 complex [77]. In this transient complex, active MST2 phosphorylates both MOB1 at T12 and T35 and LATS1 at T1079 in HM. Phosphorylation of MOB1 NTE then triggers the release of the pMOB1–LATS1 complex with T1079 already phosphorylated. Thus, MOB1 acts as a dynamic scaffold to transiently bring MST2 and LATS1 kinases into close proximity, enhancing LATS1 T1079 phosphorylation by MST2 (Figure 4). More importantly, pMOB1 retains the open conformation and acts in concert with the LATS1 p-HM to allosterically promote LATS1 autophosphorylation at S909 in its T-loop, leading to full kinase activation (Figure 4). This mode of interdependent, multistep phosphorylation events by upstream kinases MST1/2 to transduce signals to downstream LATS1/2 ensures signaling specificity.

It is not clear why MOB1 only transiently brings upstream kinases MST1/2 to downstream kinases LATS1/2, however. The timely release of MST1/2 from pMOB1 could facilitate MST1/2 binding to multiple MOB1 proteins to enhance the kinase activation of LATS1/2 and/or facilitate YAP/TAZ binding to the pMOB1–LATS1/2 complex during Hippo signaling.

## Feedback inhibition of MST1/2 activation by STRIPAK^SLMAP^

How MST1/2 activation is coupled to upstream signals remains a major unresolved question. A related puzzle is that MST1/2 form homodimers and undergo constitutive autophosphorylation *in vitro*, and yet, the vast majority of MST1/2 molecules are unphosphorylated at the T-loop *in vivo* when the Hippo pathway is off [52]. This dichotomy strongly suggests that MST1/2 activation is antagonized by phosphatases. PP2A is a major serine/threonine phosphatase with myriad functions and forms many distinct complexes with regulatory subunits. Recent proteomic and genetic studies have implicated a particular PP2A phosphatase complex, called striatin-interacting phosphatase and kinase (STRIPAK), as a negative regulator of the Hippo pathway [84–86]. In human cells, MST1/2 interacts with the adaptor protein SLMAP (sarcolemmal membrane-associated protein), which links STRIPAK to MST1/2 through phosphorylation-dependent association [87,88].

**Figure 5:**
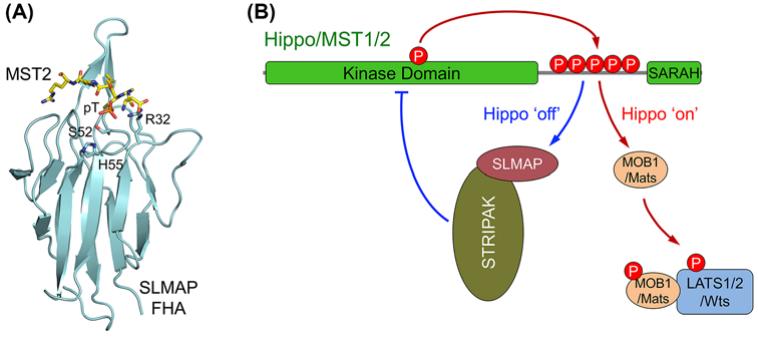
Regulation of Hippo/MST1/2 activation during Hippo signaling (**A**) Ribbon diagram of SLMAP FHA bound to a pMST2. SLMAP FHA is colored cyan. The pMST2 peptide is shown as yellow sticks. (**B**) Model for the antagonistic regulation of Hippo signaling by autophosphorylated Hippo/MST1/2 linker.

As discussed above, MST1/2 undergo constitutive autophosphorylation at multiple residues in their linker *in vitro* and in human cells, which recruits MOB1 to MST1/2 to promote Hippo pathway activation. Strikingly, three phospho-residues in the MST2 linker (pT325, pT336, and pT378) redundantly bind to the N-terminal forkhead-associated (FHA) domain of SLMAP [89,90]. The crystal structures of SLMAP FHA alone or bound to the pMST2 have revealed the structural basis of p-MST2 binding by SLMAP [89] (Figure 5A). SLMAP FHA domain adopts a typical β sandwich fold containing two large β sheets. Binding of the pMST2 peptide does not alter the conformation of the FHA domain. The pT378 and M379 residues of MST2 bind at a highly positively charged pocket and an adjacent hydrophobic pocket on SLMAP FHA, respectively. The MST2 3TA mutant with three phospho-residues mutated to alanine (T325A/T336A/T378A) is constitutively phosphorylated at pT180 in unstimulated human cells. Thus, linker phosphorylation inhibits MST2 kinase activation in a feedback mechanism. This result is consistent with earlier findings that truncation of the MST1/2 linker increases MST1/2 kinase activity [43].

SLMAP binding to the phospho-MST2 linker recruits STRIPAK to MST2 and promotes PP2A-mediated dephosphorylation of pT180. The SLMAP knockout (KO) MCF10A cells created with CRISPR-Cas9 showed that MST2 pT180 and downstream LATS1/YAP phosphorylation increased in these cells without contact inhibition [89]. Deletion of SLMAP or several core components of STRIPAK leads to increased phosphorylation at the T-loop of Hippo/MST1/2 in flies and human cells [89,91]. These results establish the STRIPAK^SLMAP^ complex as a key negative regulator of the Hippo pathway in human cells.

Interestingly, MOB1 and SLMAP bind to the pT378M site with similar affinities, suggesting that MOB1 contributes to MST1/2 activation through competing with SLMAP for MST1/2 binding [89]. Furthermore, it has recently been shown that autophosphorylation of the linker in Hippo (the fly homolog of MST1/2) creates multiple docking sites for SLAMP, which recruit STRIPAK to inactivate Hippo in *Drosophila* [90]. In contrast, autophosphorylation of the Hippo linker at distinct sites can recruit Mats (the fly homolog of MOB1) to promote Hippo signaling. Therefore, autophosphorylation of multiple sites at the Hippo/MST1/2 linker acts as an evolutionarily conserved molecular rheostat to fine-tune Hippo signaling through engaging antagonistic mechanisms (Figure 5B).

Inactivation of STRIPAK^SLMAP^ leads to spontaneous activation of the Hippo pathway without upstream signals in both fly and human cells. Due to its profound effects on MST1/2 kinase activity, the STRIPAK^SLMAP^ PP2A complex may represent a key signaling node to integrate diverse physiological signals to regulate the Hippo signaling network during animal development. For example, Toll receptor signaling has been reported to activate the Hippo pathway by phosphorylation and degradation of Cka, a core component of the *Drosophila* STRIPAK complex [92]. Future studies are needed to elucidate the physiological roles of STRIPAK^SLMAP^ in the context of Hippo pathway regulation.

## SAV1 antagonizes STRIPAK^SLMAP^ to activate MST1/2

**Figure 6:**
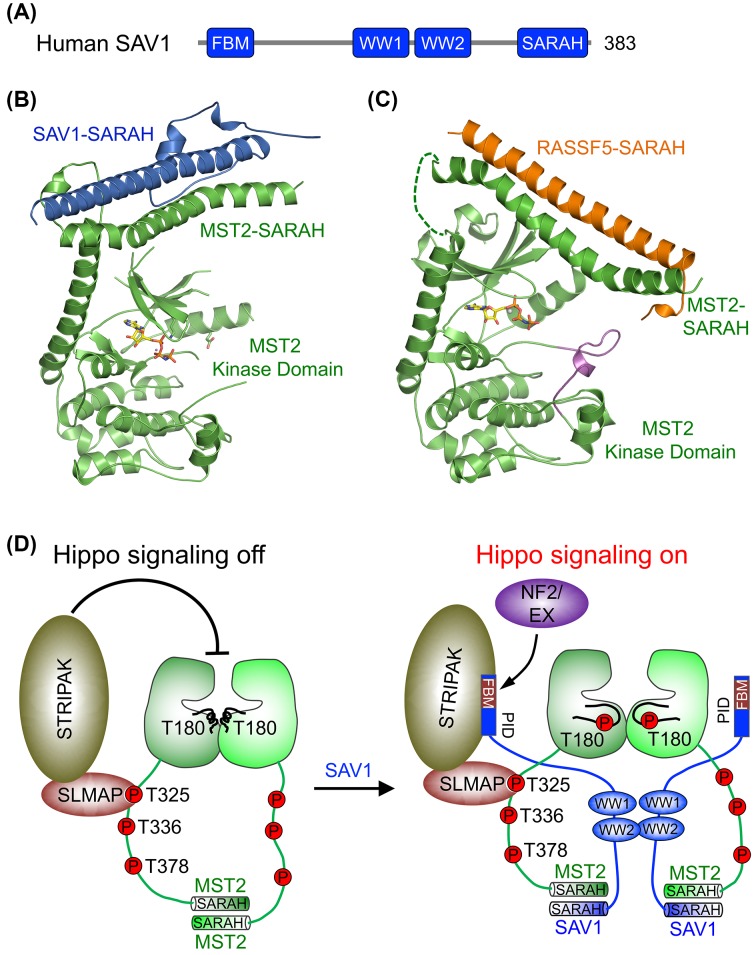
Regulation of MST2 activation by SAV1 (**A**) Domain organization of human SAV1. (**B**) Cartoon drawing of the crystal structure of the MST2–SAV1 complex (PDB ID: 6AO5). MST2 is colored green and SAV1 is colored blue. The activation loop is disordered. AMP-PNP is shown as yellow sticks. (**C**) Cartoon drawing of the crystal structure of the MST2–RASSF5 complex (PDB ID: 4LGD). RASSF5 is colored orange and the activation loop is colored magenta. (**D**) Model for SAV1-dependent MST2 activation during Hippo signaling. MST2–SAV1 forms a heterotetramer through SAV1 WW domains. The N-terminal phosphatase inhibitory domain (PID) of SAV1 including the FBM directly inhibits PP2A and prevents pT180 dephosphorylation by STRIPAK^SLMAP^, which is recruited to MST2 through phospho-residues in the MST2 linker. NF2/EX or other FERM-containing proteins may regulate the PP2A-blocking activity of SAV1.

MST1/2 activation requires its binding to SAV1 *in vivo*, as MST1/2 are not activated in the keratinocytes from SAV1 KO mice [93]. SAV1 contains an N-terminal flexible region which contains the FERM-binding motif (FBM) that binds to the FERM domain of NF2 or EX, two WW domains in tandem, and a C-terminal SARAH domain (Figure 6A) [94]. MST1/2 and their regulators, SAV1 and RASSFs, are the only components in the Hippo pathway that contain a C-terminal SARAH domain. Like RASSFs, SAV1 interacts with MST1/2 through SARAH domains [95]. RASSF binding and SAV1 binding to MST1/2 are mutually exclusive [47,50]. Binding of SAV1 to MST1/2 promotes Hippo signaling through the formation of a MST2–SAV1 heterotetramer. Binding of MST1/2 increases SAV1 stability and enables phosphorylation of SAV1 by MST1/2, although the functional consequence of this phosphorylation event is not clear [7,95–98]. How RASSFs and SAV1 regulate MST1/2 activation by forming different SARAH domain-dependent complexes has remained a mystery until recently.

The crystal structure of MST2 bound to the SARAH domain of SAV1 shows, surprisingly, that MST2–SAV1 does not form the expected heterotetramer through a four-helix bundle formed by their SARAH domains (Figure 6B) [89]. Instead, SAV1 SARAH and MST2 SARAH form a heterodimer, similar to that formed by RASSF5 SARAH and MST2 SARAH (Figure 6C). A recent crystal structure of the *Drosophila* Salvador SARAH–Hippo SARAH complex reveals a similar heterodimeric assembly of the SARAH domains [99]. The SAV1- and RASSF5-binding surfaces of MST2 SARAH are virtually identical, readily explaining the mutually exclusive binding of SAV1 and RASSF5 to MST2. Thus, the structural analyses support the notion that RASSFs antagonize the SAV1 function by competing for MST1/2 binding. In addition, *Drosophila* Rassf has been shown to bring STRIPAK to Hippo to suppress its kinase activity [84]. More studies are needed to elucidate the precise roles of the SLMAP- and RASSF-mediated recruitment of STRIPAK to Hippo/MST1/2 in tissue homeostasis.

MST1/2 autoactivation requires two kinase domains to be in close proximity for efficient trans-autophosphorylation, however, SAV1 and MST2 only form a heterodimer through the SARAH interaction. Intriguingly, the second atypical WW domain of SAV1 can form a dimer in solution [89,100]. Thus, upon Hippo activation, SAV1 binds to MST2 and forms a SAV1–MST2 heterodimer through their SARAH domains. SAV1 WW domains further dimerize to form a SAV1–MST heterotetramer. In the context of this heterotetramer, the two kinase domains of MST2 can undergo trans-autophosphorylation at T180 and activation.

Strikingly, the N-terminal tail of SAV1 directly binds to PP2A and inhibits its phosphatase activity, suggesting that SAV1 stimulates MST2 activation through antagonizing STRIPAK^SLMAP^-mediated dephosphorylation of MST2 pT180 [89]. STRIPAK^SLMAP^ and the SAV1–MST2 heterotetramer can form a supercomplex through SLMAP FHA binding to the activated MST2. When Hippo signaling is turned on, the molecular interactions amongst MST2, SAV1, and STRIPAK^SLMAP^ position the N-terminal phosphatase-inhibitory domain (PID) of SAV1 for direct binding to and inhibition of the PP2A catalytic core, thus protecting pT180 from dephosphorylation. Deletion of SLMAP bypasses the requirement of SAV1 in Hippo pathway activation in normal growth conditions, providing conclusive evidence for the antagonism between SAV1 and STRIPAK^SLMAP^ *in vivo* [89]. Therefore, SAV1 promotes MST1/2 activation through forming an MST1/2–SAV1 heterotetramer that permits MST1/2 autoactivation, and through antagonizing PP2A-mediated dephosphorylation of the activation loop of MST1/2 (Figure 6D). Future studies on the reconstitution and structure of the SAV1–MST1/2–STRIPAK^SLMAP^ supercomplex will further define the molecular mechanism and structural basis of the opposing regulation of MST2 by SAV1 and STRIPAK^SLMAP^.

SAV1 also promotes MST1/2 activation and Hippo signaling through other mechanisms [98,101]. For example, MST1/2 can be targetted to the plasma membrane by SAV1, whereas NF2 promotes the plasma membrane association of LATS1 [37]. The co-localization of MST1/2 and LATS1/2 at the membrane facilitates MST1/2-dependent phosphorylation and activation of LATS1/2. In addition, SAV1 has been proposed to recruit substrates to MST1/2 for efficient phosphorylation [6,7,96,101]. The discovery that SAV1 antagonizes STRIPAK^SLMAP^ to promote MST1/2 activation further implies that SAV1 targets the active MST1/2 to appropriate substrates. On the other hand, the membrane protein SLMAP has been shown to predominantly localize to the mitochondria and endoplasmic reticulum (ER) [86,90,102–104]. It is possible that SLMAP recruits MST1/2 to certain subcellular locations for STRIPAK action.

How the SAV1–STRIPAK^SLMAP^ antagonism is regulated by upstream signals is unknown. Intriguingly, the N-terminal PID of SAV1 encompasses the FBM that binds to FERM domains. Deletion of SAV1 FBM reduces its ability to activate MST2 in human cells [89]. This finding suggests a plausible connection between the cytoskeletal complex NF2–EX and MST1/2 activation. It is possible that PID-mediated PP2A inhibition is regulated by upstream signals due to the presence of FBM in SAV1 PID. SAV1 FBM is known to bind to FERM-containing proteins, such as NF2 and EX, which have been reported to activate MST1/2 in cardiomyocytes and peripheral nerve fibroblasts, respectively [105,106]. Moreover, the FBM-deleted SAV1 mutant activates MST2 much less efficiently than does SAV1 WT. These findings suggest that NF2/EX may regulate PP2A inhibition of MST1/2 through engaging the N-terminal PID of SAV1. Alternatively, they may bind and re-organize other core components of STRIPAK after their recruitment by SAV1 FBM. Intriguingly, when the Hippo pathway is off, NF2 adopts a closed conformation, in which its FERM domain is autoinhibited and cannot bind to FBMs [107]. When the Hippo pathway is on, NF2 adopts an open conformation, in which its FERM domain binds to the FBM of LATS1/2 and recruits LATS1/2 to the membrane for phosphorylation by MST1/2 [37]. Oncogenic NF2 mutations are believed to affect the open-closed conformational change in NF2, thus inhibiting Hippo signaling [37,107]. It will be interesting to test whether NF2 or other FERM-containing proteins regulate the PP2A-blocking function of SAV1 in similar ways. Future studies are needed to reveal how upstream regulators affect functional antagonism between SAV1 and STRIPAK^SLMAP^ on the regulation of Hippo pathway activation.

## Conclusion

**Figure 7:**
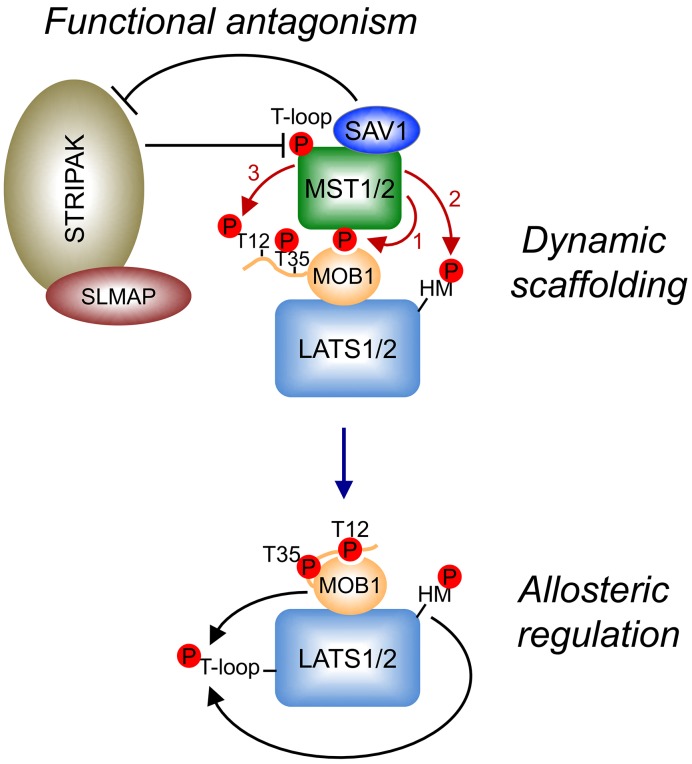
Mechanisms of the core Hippo pathway regulation Key phosphorylation events are indicated by dark red arrows

The Hippo pathway controls tissue growth and homeostasis in multicellular organisms. A large collection of studies has identified many components of this pathway and revealed their cellular functions. Diverse upstream signals activate the central MST–LATS kinase cascade in the Hippo pathway, which regulates the YAP/TAZ–TEAD transcription module to alter transcription. Recent structural and mechanistic studies have established the molecular mechanisms for the key phosphorylation-dependent events during the activation of the core MST1/2–LATS1/2 kinase cascade. These studies have established the dynamic scaffolding and allosteric roles of MOB1 in LATS1/2 activation by MST1/2 and the functional antagonism between SAV1 and STRIPAK^SLMAP^ in regulating MST1/2 activation (Figure 7). These studies have further highlighted the importance of multisite autophosphorylation by MST1/2 in fine-tuning the signaling strength and robustness of the Hippo pathway.

Although great progress has been made in understanding the regulation of the core kinase cascade, many outstanding questions remain in this active field. For example, how do upstream signals impinge on the SAV1-STRIPAK antagonism through NF2–EX and other cytoskeletal elements to regulate Hippo activation? What are the other physiological signals that converge on the STRIPAK^SLMAP^ PP2A complex to regulate Hippo signaling? How do p-MOB1 and p-HM of LATS1/2 work in concert to allosterically activate LATS1/2 kinases and promote downstream signaling? Answering these questions will significantly advance our understanding of the regulation of the Hippo pathway under physiological and pathological conditions, and help to guide the development of targetted therapeutic interventions to treat human diseases caused by dysregulated Hippo signaling.

## Abbreviations

HM: hydrophobic motif
HS: hydrophobic sequence
KO: knockout
LATS1/2: large tumor suppressor 1 and 2
MBD: MOB-binding domain
Mer: Merlin
MOB1: Mps One Binder 1
MST1/2: mammalian Ste20-like 1 and 2
NF2: neurofibromatosis type 2
NTE: N-terminal extension
PID: phosphatase-inhibitory domain
pMST2: pT378 MST2 linker peptide
pT: p-threonine
RASSF: Ras-association domain family
SARAH: Salvador/RASSF/Hippo
SAV1: Salvador
SLMAP: sarcolemmal membrane-associated protein
STRIPAK: striatin-interacting phosphatase and kinase
Tao-1: Tao kinase 1
TAZ: transcriptional co-activator with PDZ-binding motif
TEAD: TEA domain protein
YAP: Yes-associated protein
